# The mediating effect of sleep disturbance on the association between hypertension and depression: a national data analysis

**DOI:** 10.1186/s40885-024-00263-y

**Published:** 2024-02-01

**Authors:** Kamaluddin Latief, Samuel Akyirem, Siriluk Sithichoksakulchai, Dieta Nurrika, Mokh. Sujarwadi, Faizul Hasan

**Affiliations:** 1 Global Health and Health Security, College of Public Health, Taipei Medical University, Taipei, Taiwan; 2 Centre for Family Welfare, Faculty of Public Health, Universitas Indonesia, Depok, Indonesia; 3 Yale School of Nursing, Yale University, New Haven, CT USA; 4 Department of Fundamental Nursing, Faculty of Nursing, Mahidol University, Bangkok, Thailand; 5 College of Nursing, Kaohsiung Medical University, Kaohsiung, Taiwan; 6 Public Health Study Program, Banten School of Health Science, South Tangerang, Indonesia; 7 Culture, Research, and Technology, The Ministry of Education, Higher Education Service Institutions (LL-DIKTI) Region IV, Bandung, Indonesia; 8 Faculty of Nursing, Universitas Jember, Jember, Indonesia; 9 Faculty of Nursing, Chulalongkorn University, Boromarajonani Srisataphat Building, 12th Floor Rama1 Road, Wang Mai, Pathum Wan, Bangkok, 10330 Thailand

**Keywords:** Sleep, Depression, Nursing home care, Nurse observation, Nurse roles, Nurse practitioners, Nurse-patient interaction, Neurology, Mental health, Medical nursing

## Abstract

**Background:**

Sleep disturbance is a common among people with hypertension. However, the mediating role of sleep disturbance in the association between hypertension and depression remains unclear. This study aims to investigate the mediating role of sleep disturbance in the association between hypertension and depression.

**Materials and methods:**

This was cross-sectional study. The data were derived from the Indonesian Family Life Survey Fifth Wave (2014–2015). We include a total of 19,138 adults’ participants with age range from 18 to 65 years old who completed response on the variable of hypertension, sleep disturbance, and depression. The mediating model analysis was processed using the PROCESS macro ins SPSS from Hayes model.

**Results:**

Depression was reported by 22% of total respondents. The group with hypertension showed a substantially higher prevalence of depression than non-hypertension group (*P* < 0.001). Hypertension had a significant overall effect on depression (β = 0.682; 95%CI 0.489 to 0.875, *P* < 0.001). The direct effect of hypertension on depression was significant (β = 0.418; 95%CI 0.244 to 0.592, *P* < 0.001) and the indirect effect that mediated by sleep disturbance was also significant (β = 0.264, 95%CI 0.174 to 0.356, *P* < 0.001). It is worth noting that sleep disturbance partially mediated the association between hypertension and depression.

**Conclusion:**

The findings of this study indicated that sleep disturbance contributed to the etiology of depression and hypertension in adult populations. Nurses should be involved in managing sleep disturbances, such as using behavioral therapy, as it may serve as both a treatment and primary prevention measure for depression and hypertension.

**Graphical Abstract:**

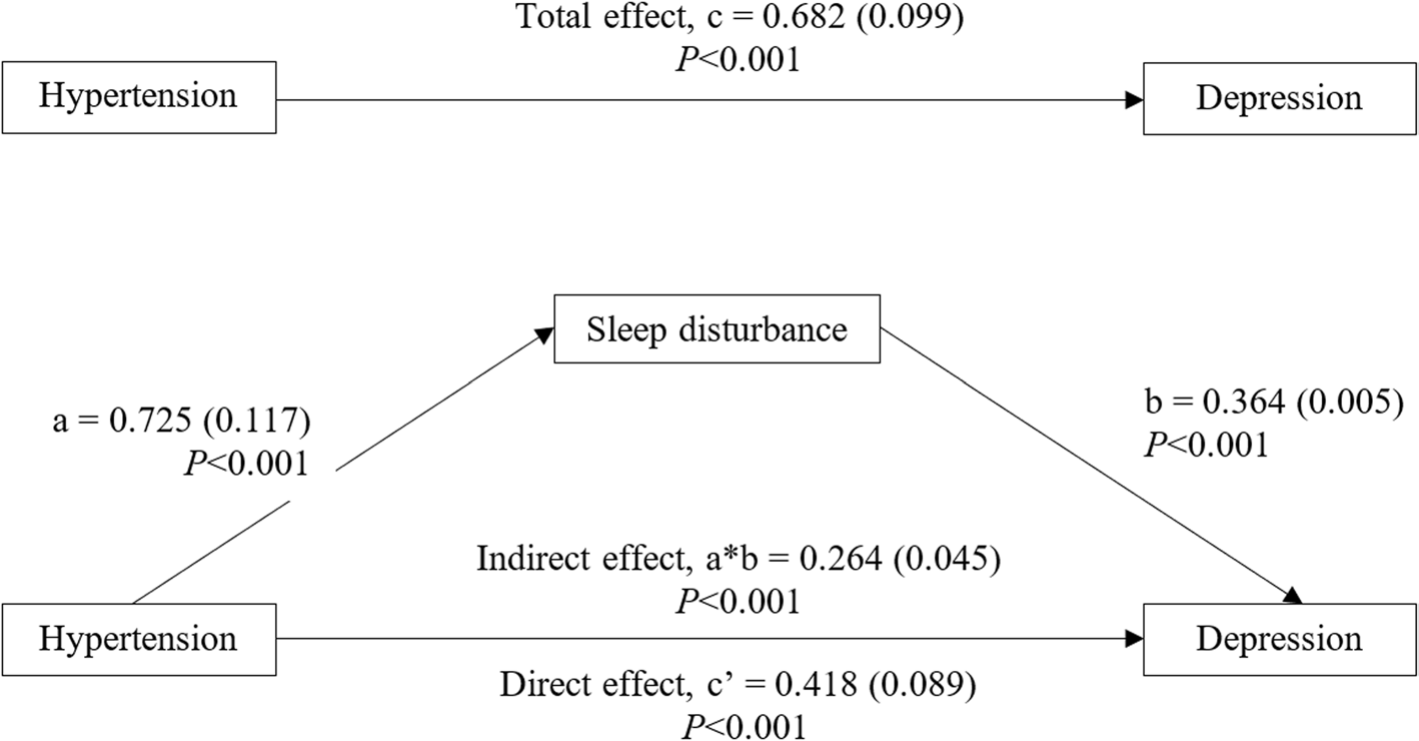

**Supplementary Information:**

The online version contains supplementary material available at 10.1186/s40885-024-00263-y.

## Background

Hypertension is a common chronic condition with global prevalence ranging from 31 to 56% [1, 2]. In Indonesia, about 8.4% of adults have been diagnosed with hypertension by a medical doctor [3]. Hypertension has been linked to elevated risk of cardiovascular comorbidities including stroke [4] and chronic kidney diseases [5, 6]. Other evidence suggests that people with hypertension are more likely to experience depression [7, 8].

Several studies have reported prevalence rates ranging from 22 to 32% [7, 9]. Previous studies suggest a bidirectional association between hypertension and depression [9, 10]. Depression may increase the risk of hypertension by activating the body’s stress responses and increasing autonomic nervous system activation [11]. On the other hand, hypertension may increase the risk for depression by causing cerebrovascular pathologies [12] and increasing the sense of hopelessness related to living with a chronic condition [13]. People who suffer from both depression and hypertension often report low quality of life [14]. In addition, they often experience sleep disturbances [15]. While, the underlying mechanism of the hypertension-depression relationship remain complex, sleep disturbance has been identified as a potential mediator [15].

Sleep disturbance has been linked to physical and mental health including cardiovascular disease and mood disorders [16]. People with sleep problem have high risk to develop hypertension [17] and depression [18]. However, there is a lack of study investigating the mediating role of sleep disturbance in the association between hypertension and depression. Hence, in this study we aim to investigate the mediating role of sleep disturbance in the association between hypertension and depression using nationally representative data from Indonesia.

## Materials and methods

### Data source and participants

The data was derived from Indonesian Family Life Survey (IFLS) fifth Wave (IFLS-5), which is a cross-sectional study, fielded in September 2014 to April 2015. The IFLS sample represents approximately 83% of the Indonesian population living in 13 of the country’s 26 provinces, covering 16,204 households and 50,148 individuals [19]. The IFLS has been conducted 5 times, in 1993, 1997, 2000, 2007, and 2014. The details of study design of the IFLS-5 have been previously described [19]. In this study, we include a total of 19,138 adults’ participants with age range from 18 to 65 years old who have completed response on the variable of hypertension, sleep disturbance, and depression.

### Ethical approval

The IFLS data are accessible to the general public. Institutional review boards at the University of Gajah Mada in Indonesia and the RAND Corporation in the United States have examined and approved the survey’s methods with the ethical clearance No.s0064-06-01-CR01 [19]. Before data collection began, all participants provided their written, informed consent.

### Measurements

#### Depression

Depression was measured using the short form Centres for Epidemiologic Studies Depression Scale (CES-D-10) [20]. CES-D-10 consist of 10 items rated on a 4-point Likert scale ranging from 0 (rarely or none of the time) to 3 (all of the time) with higher score indicating more depressive symptoms. A total score of 10 or higher is indicative of having depression. Previous study revealed that this scale has acceptable validity and reliability [19].

#### Hypertension

Previous studies using IFLS data indicate that hypertension, as measured by blood pressure, covers approximately 40% of participants [21, 22], while hypertension using self-reported measurements covers around 80% of participants [21]. Therefore, in this study, hypertension was assessed using self-report, measured with the question, “Has a doctor, paramedic, or nurse ever told you that you had hypertension?”.

#### Sleep disturbance

Sleep disturbance was measured using the combination of five items of Patient-Reported Outcomes Measurement Information System (PROMIS) sleep disturbance measure [23] and five items of PROMIS sleep impairment measure [24]. Previous study reported that the scale has good Cronbach’s alpha of 0.82 [25]. Higher scores on the scales indicate more sleep disturbance with a cut-off point 11 or higher indicating of having sleep disturbance [25].

#### Lifestyle and comorbidity condition

Smoking status was measured and classified into three groups (never, quitters and current tobacco users) [19]. Physical activity was measured using the short version of International Physical Activity Questionnaire (IPAQ) for the last 7 days (IPAQ-S7S) [26]. It is divided into three level of low, moderate, and high intensity physical activity. Additionally, for the outpatient care were assessed using single item questionnaire [19].

For comorbidity conditions (diabetes mellitus, tuberculosis, asthma, lung condition, heart attack, liver disease, stroke, cancer, high cholesterol, kidney disease, stomach or other digestive disease, and psychiatric problem). It was measured using single item question such as ‘Has a doctor/paramedic/nurse/midwife ever told you that you had…? to which participants answered “yes” or “no” [19].

#### Demographic

In addition, demographic variable including age, gender, marital status, attending school, and education level were added in this study [19].

### Statistical analyses

At the beginning, we used STATA software for all data processing. In the second stage, we used SPSS software version 29.0 to perform all statistical analyses (IBM, Armonk, NY, USA). A two-tailed of *P* < 0.05 was considered as a statistically significant level. To analyse the differences in the baseline characteristics between the 2 groups, we used the chi-squared or Fisher’s exact test for categorical variables and independent t-tests for continuous variables. The correlation between main variable (hypertension, sleep disturbance, and depression) were measured using Pearson and Spearman correlation. Univariate and multivariate linear regression analyses were also performed. Finally, the mediating model analysis was processed using the PROCESS macro ins SPSS from Hayes model [27].

## Result

### Study characteristic

**Fig. 1:**
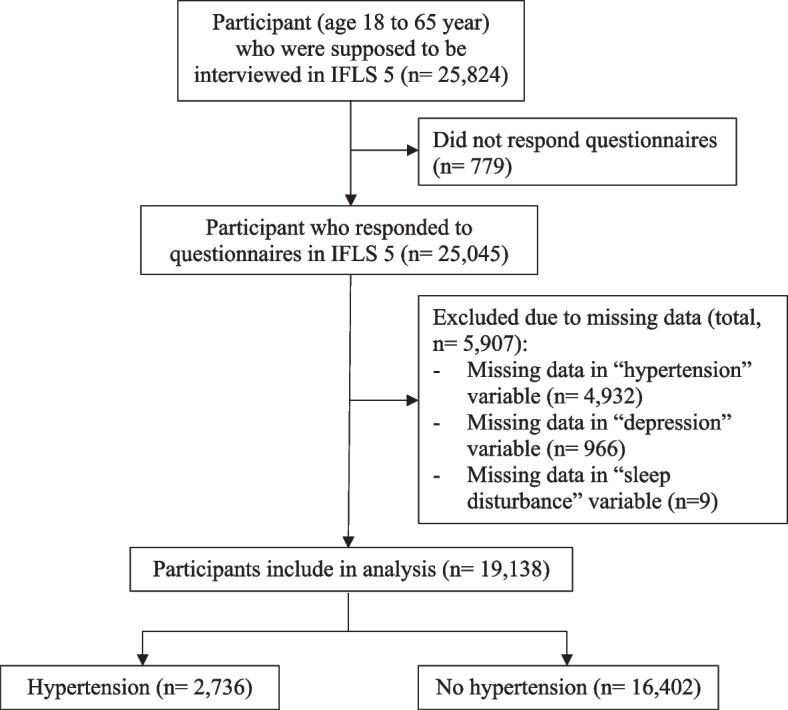
Participant flow chart. *n* = number of participants

Figure 1 depicted the participants’ flow diagram. In total, there were 25,824 adult participants were screened for eligibility. Of these, a total of 6,686 participants were excluded, with 779 not responding to questionnaires and 5,907 having missing data. This included missing data in the ‘hypertension’ variable (*n* = 4,932), ‘depression’ variable (*n* = 966), and ‘sleep disturbance’ variable (*n* = 9). Finally, a total of 19,138 participants were included in the main analysis. Hypertension was confirmed for 2,736 (14.3%) individuals.

Demographic comparisons between patients with hypertension (*n* = 2,736) and without hypertension (*n* = 16,402) were presented in Table 1. The prevalence of hypertension varies across age groups, with rates of 6.5% for the 18–34 age group, 13.1% for the 35–49 age group, and 25.9% for the 50–65 age group. Most of the participants were married (84%) and 96% of them have attended the school.

**Table 1:** Demographic comparison of participants

Variables	Total (*n* = 19,138)		Missing data		Hypertension (*n* = 2736)		No hypertension (*n* = 16,402)		* p*-value
	n	(%)	n	(%)	n	(%)	n	(%)	
Age									< 0.001
18–34 years	6410	(33.5)			418	(15.3)	5992	(36.5)	
35–49 years	7635	(39.9)			998	(36.5)	6637	(40.5)	
50–65 years	5093	(26.6)			1320	(48.2)	3773	(23)	
Gender			1	(0.0)					< 0.001
Female	10,469	(54.7)			1817	(66.4)	8652	(52.7)	
Male	8668	(45.3)			919	(33.6)	7749	(47.2)	
Marital status			4	(0.0)					< 0.001^a^
Single	1355	(7.1)			81	(3)	1274	(7.8)	
Married	16,058	(83.9)			2263	(82.7)	13,795	(84.1)	
Separated	102	(0.5)			12	(0.4)	90	(0.5)	
Divorced	487	(2.5)			68	(2.5)	419	(2.6)	
Windowed	1128	(5.9)			310	(11.3)	818	(5)	
Cohabitate	4	(0.0)			2	(0.1)	4	(0.0)	
Have you ever attended/ are you attending school?			2	(0.0)					< 0.001
Yes	18,365	(96)			2571	(94)	15,794	(96.3)	
No	771	(4)			165	(6)	606	(3.7)	
Education level			773	(4)					< 0.001
University level	1891	(9.9)			220	(8)	1671	(10.2)	
Below university level	17,247	(90.1)			2516	(92)	14,731	(89.8)	

Continuous variable was performed by using independent t-test,

Categorical variables were performed by using chi-square test or ^a^Fisher’s exact test

The comparison of lifestyle and comorbidity conditions were presented in Table 2. In total 37% of included participants were smoker. The hypertension group had higher sleep disturbance compared to the non-hypertension group. There is a significant difference between the two groups in terms of physical activity (vigorous and low activity levels) (*P* < 0.001 and *P* = 0.001, respectively). Other details of the variable can be seen in Table 2.

**Table 2:** Lifestyle and comorbid comparison of participants

Variables	Total (*n* = 19,138)		Missing data		Hypertension (*n* = 2736)		No hypertension (*n* = 16,402)		* p*-value
	n	(%)	n	(%)	n	(%)	n	(%)	
Smoking									< 0.001
Yes	7065	(36.9)			748	(27.3)	6317	(38.5)	
No	12,073	(63.1)			1988	(72.7)	10,085	(61.5)	
Sleep disturbance									< 0.001
Yes	2723	(14.2)			468	(17.1)	2255	(13.7)	
No	16,415	(85.8)			2268	(82.9)	14,147	(86.3)	
Depression									< 0.001
Yes	4068	(21.3)			679	(24.8)	3389	(20.7)	
No	15,070	(78.7)			2057	(75.2)	13,013	(79.3)	
Physical activity during last 7 days									
Vigorous activities									< 0.001
Yes	4216	(22)			484	(17.7)	3732	(22.8)	
No	14,922	(78)			2252	(82.3)	12,670	(77.2)	
Moderate activities									0.68
Yes	10,908	(57)			1549	(56.6)	9359	(57.1)	
No	8230	(43)			1187	(43.4)	7043	(42.9)	
Low activities									0.001
Yes	13,545	(70.8)			2009	(73.4)	11,536	(70.3)	
No	5593	(29.2)			727	(26.6)	4866	(29.7)	
Did you have outpatient care last 4 weeks?			8	(0.0)					< 0.001
Yes	3716	(19.4)			868	(31.7)	2848	(17.4)	
No	15,414	(80.5)			1868	(68.3)	13,546	(82.6)	
Comorbidity									
Diabetes mellites									< 0.001
Yes	538	(2.8)			233	(8.5)	305	(1.9)	
No	18,600	(97.2)			2503	(91.5)	16,097	(98.1)	
Tuberculosis									0.35
Yes	187	(1)			31	(1.1)	156	(1)	
No	18,951	(99)			2705	(98.9)	16,246	(99)	
Asthma									< 0.001
Yes	513	(2.7)			118	(4.3)	395	(2.4)	
No	18,625	(97.3)			2618	(95.7)	16,007	(97.6)	
Other lung condition									0.48
Yes	332	(1.7)			52	(1.9)	280	(1.7)	
No	18,806	(98.3)			2684	(98.1)	16,122	(98.3)	
Heart attack									< 0.001
Yes	384	(2)			153	(5.6)	231	(1.4)	
No	18,754	(98)			2583	(94.4)	16,171	(98.6)	
Liver disease									0.54
Yes	201	(1.1)			25	(0.9)	176	(1.1)	
No	18,937	(98.9)			2711	(99.1)	16,226	(98.9)	
Stroke									< 0.001
Yes	135	(0.7)			95	(3.5)	40	(0.2)	
No	19,003	(99.3)			2641	(96.5)	16,362	(99.8)	
Cancer									0.01
Yes	140	(0.7)			31	(1.1)	109	(0.7)	
No	18,998	(99.3)			2705	(98.9)	16,293	(99.3)	
High cholesterol									< 0.001
Yes	1031	(5.4)			438	(16)	593	(3.6)	
No	18,107	(94.6)			2298	(84)	15,809	(96.4)	
Kidney disease									< 0.001
Yes	303	(1.6)			83	(3)	220	(1.3)	
No	18,835	(98.4)			2653	(97)	16,182	(98.7)	
Stomach or other digestive disease									< 0.001
Yes	2499	(13.1)			503	(18.4)	1996	(12.2)	
No	16,639	(86.9)			2233	(81.6)	14,406	(87.8)	
Psychiatric problem									0.04
Yes	32	(0.2)			9	(0.3)	23	(0.1)	
No	19,106	(99.8)			2727	(99.7)	16,379	(99.9)	

Categorical variables were performed by using chi-square test

### Depression

The depression was reported in 22% of the total sample. The group with hypertension had a significantly higher depression prevalence compared to the non-hypertension group (see Table 2, *P* < 0.001). Supplementary Table S1 presented the response of participants who endorse each of the depression questionnaires.

### Association between the hypertension, sleep disturbance, and depression

Supplementary Table S2 shows the association between the hypertension, sleep disturbance, and depression. The hypertension variable had a significant positive correlation with depression (*r* = 0.05, *P* < 0.001). The hypertension variable also has a significant positive correlation with sleep disturbance (*r* = 0.05, *P* < 0.001). Similarly sleep disturbance variable also has a significant positive correlation with depression (*r* = 0.44, *P* < 0.001).

The bivariate and multiple linear regression results are displayed in Table 3. In the unadjusted model, all predictors show a significant positive association with the target variable. When hypertension and sleep disturbance are combined as predictors in Model 1, the association remains significantly positive. In Model 2, all predictors also exhibit a significant positive association with the outcome variables.

**Table 3:** Associations from multiple linear regression models of hypertension with sleep disturbance, and depression

Predictor	Target variable	Unadjusted					Model 1			Model 2
		β^a^	β^s^	(95% CIs)	β^a^	β^s^	(95% CIs)	β^a^	β^s^	(95% CIs)
Hypertension	Depression	0.68	0.05	(0.49 to 0.88)	0.94	0.07	(0.79 to 1.19)	0.60	0.04	(0.40 to 0.80)
Hypertension	Sleep disturbance	0.73	0.05	(0.49 to 0.96)	0.88	0.05	(0.68 to 1.17)	0.50	0.03	(0.27 to 0.73)
Hypertension	Depression	0.42	0.03	(0.24 to 0.59)	0.66	0.05	(0.47 to 0.84)	0.47	0.03	(0.29 to 0.66)
Sleep disturbance		0.36	0.43	(0.35 to 0.38)	0.37	0.43	(0.35 to 0.37)	0.37	0.44	(0.36 to 0.39)

model 1 = adjusted for variables in Table 1, model 2 = adjusted for variables in Tables 1 and 2

β^a^ = Unstandardized coefficients, β^s^ = Standardized coefficients, CI = Confidence intervals

### Mediating effect of sleep disturbance on the association between hypertension and depression

**Fig. 2:**
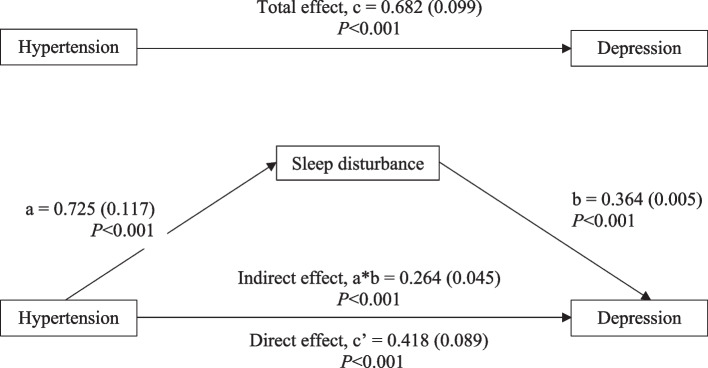
Mediation Analysis Results. The data were presented in Beta coefficient (standard error)

The simple mediating analysis indicated that sleep disturbance partially mediated the association between hypertension and depression. As shown in Fig. 2 and supplementary Table 3, hypertension was positively associated with sleep disturbance (a = 0.725), and sleep disturbance was positively associated with depression (b = 0.364). Based on 5,000 bootstrap resamples, the bootstrap confidence interval for the indirect effect (ab = 0.264) was entirely above zero, ranging from 0.174 to 0.356. The total effect of hypertension on depression was significant (β = 0.682; 95% CI 0.489 to 0.875, *P* < 0.001), comprising a direct effect (β = 0.418; 95% CI 0.244 to 0.592, *P* < 0.001) and an indirect effect (β = 0.264; 95% CI 0.174 to 0.356, *P* < 0.001).

## Discussion

To the best of our knowledge, this is the first study investigating the mediating effect of sleep disturbance in the association between hypertension and depression. This study highlights that sleep disturbance becomes partial mediation in the association between hypertension and depression. Because the methodology is rigorous and we use a big sample size, hence our study should be considered.

Although the pathological mechanism of sleep disturbance after hypertension was complex. However, it can develop as a result of hypertension due to factors such as persistent physiological stress [28], increased sympathetic nervous system activity causing alertness [29, 30], disrupted nighttime blood pressure patterns [31], potential sleep-affecting medication side effects [32], psychological impact from hypertension management [33], and endothelial dysfunction [34, 35], all of which contribute to sleep disruptions. Effective hypertension treatment, lifestyle changes [36], and stress reduction measures are critical for sleep disturbance [37]. If sleep problems persist, it is critical to consult a healthcare practitioner, as treating hypertension may improve sleep quality [37–39].

In this study, sleep disturbance is observed in approximately 17% of the hypertension group and 13.7% of the non-hypertension group. Consistent with previous studies, sleep disturbances such as obstructive sleep apnea (OSA) and insomnia are prevalent following hypertension [32, 40, 41]. Evidence suggests that OSA becomes a significant risk factor for hypertension [42]. Numerous pathways, including endothelial impairment [43, 44], oxidative stress [45, 46], inflammation [47, 48], and sympathetic activation [49, 50], are generally acknowledged as ways in which OSA leads to the development of hypertension. Of note, this study reveals that sleep disturbance partially mediates the association between hypertension and depression. It indicates that targeting depression treatment after hypertension should also consider sleep disturbance.

We found that the prevalence of depression following hypertension in our study is 25% which is higher than in the non-hypertension group (21%, see Table 2). In line with previous studies, the prevalence of depression was ranging from 22 to 32% following hypertension [7, 9] and 13–17% in the general population [51, 52]. Because the presence of depression affects the health-related quality of life [14]. Hence, it is important for clinicians and researchers to implement the best treatment approach for depression.

### Strengths and limitations

To the best of our knowledge, this study possesses several strengths. First, this study was national representativeness of the data since the study population was taken from participants across Indonesia. Second, all interviewers for the IFLS were trained to understand the methodology and the content of the questionnaire.

This study highlights several limitations. First, the data related severity of hypertension, subtype, and type of medication is not available. Second, potential confounders could not obtain such use hypnotic used, dietary factors, and environmental factors, which may threaten the internal validity. Third, the data related to blood pressure and medication for hypertension is unable to be provided. Fourth, the data related to hypertension was self-reported, which may cause information bias.

## Conclusion

The result of this study suggested that sleep disturbance plays a role in the etiology of hypertension and depression in adult populations. The management of sleep disturbance could potentially serve as treatment and primary prevention for depression in these populations. Behavioral therapy could be implemented to reduce sleep disturbance.

### Supplementary Information

**Additional file 1: Supplementary Table S1.** Depression items: the percentages of participants who endorse each response (*N* = 19138).

**Additional file 2: Supplementary Table S2.** The correlation between main variables (*N* = 19138).

**Additional file 3: Supplementary Table S3.** Mediating analysis.

## Data Availability

The datasets used and/or analyzed during the current study are available from the corresponding author on reasonable request.

## Abbreviations

IFLS: Indonesian Family Life Survey
CES-D-10: Centers for Epidemiologic Studies Depression Scale
PROMIS: Patient-Reported Outcomes Measurement Information System
IPAQ: International Physical Activity Questionnaire
OSA: Obstructive sleep apnea
