# Supplementary material for: The mediating effect of sleep disturbance on the association between hypertension and depression: a national data analysis
Source: Clin Hypertens. 2024 Feb 1;30:5. doi: 10.1186/s40885-024-00263-y (PMC10832256; doi:10.1186/s40885-024-00263-y)
Supplement: Supplementary file 1 — Additional file 1: Supplementary Table S1. Depression items: the percentages of participants who endorse each response (N = 19138). [file 40885_2024_263_MOESM1_ESM.docx]

Supplementary Table S1. Depression items: the percentages of participants who endorse each response (N = 19138).

| **Item** | **Questions** | **Rarely or none (≤1 day)** | **Some days (1-2 days)** | **Occasionally (3-4 days)** | **Most of the time (5-7 days)** |
| --- | --- | --- | --- | --- | --- |
| 1 | I was bothered by things that usually don't bother me | 66.5 | 15.5 | 13.9 | 4.1 |
| 2 | had trouble concentrating in what I was doing | 66.4 | 15.9 | 13.8 | 3.9 |
| 3 | I felt depressed | 75.7 | 11.5 | 9.1 | 3.6 |
| 4 | I felt everything I did was an effort | 57.9 | 15 | 14.8 | 12.3 |
| 5 | I felt hopeful about the future | 11.9 | 16.7 | 22.6 | 48.9 |
| 6 | I felt fearful | 70.9 | 13.1 | 11.1 | 4.9 |
| 7 | My sleep was restless | 62.7 | 11.9 | 14.7 | 10.7 |
| 8 | I was happy | 8.6 | 17.4 | 28.4 | 45.7 |
| 9 | I felt lonely | 82.6 | 7.2 | 7.1 | 3 |
| 10 | I could not get going | 75.1 | 10 | 11 | 3.9 |

Item 1-4,6-7,9-10 are negative experiences that were scored were scored from 0 (rarely or none of the time) to 3 (most or all of the time (5–7 days)); item 5 and 8 are positive experiences that were scored were scored from 3 (rarely or none of the time) to 0 (most or all of the time (5–7 days)).
