# Supplementary material for: The mediating effect of sleep disturbance on the association between hypertension and depression: a national data analysis
Source: Clin Hypertens. 2024 Feb 1;30:5. doi: 10.1186/s40885-024-00263-y (PMC10832256; doi:10.1186/s40885-024-00263-y)
Supplement: Supplementary file 2 — Additional file 2: Supplementary Table S2. The correlation between main variables (N = 19138). [file 40885_2024_263_MOESM2_ESM.docx]

Supplementary Table S2. The correlation between main variables (N = 19138).

|  | Hypertension | Sleep disturbance | Depression |
| --- | --- | --- | --- |
| Hypertension | 1 | 0.05** | 0.05** |
| Sleep disturbance | 0.05** | 1 | 0.44** |
| Depression | 0.05** | 0.44** | 1 |

***P*<0.001
