# Supplementary material for: The mediating effect of sleep disturbance on the association between hypertension and depression: a national data analysis
Source: Clin Hypertens. 2024 Feb 1;30:5. doi: 10.1186/s40885-024-00263-y (PMC10832256; doi:10.1186/s40885-024-00263-y)
Supplement: Supplementary file 3 — Additional file 3: Supplementary Table S3. Mediating analysis. [file 40885_2024_263_MOESM3_ESM.docx]

Supplementary Table S3. Mediating analysis

|  |  | M (Sleep disturbance) | | | |  | Y (Depression) | | | |
| --- | --- | --- | --- | --- | --- | --- | --- | --- | --- | --- |
| Antecedent |  | β^A^ | SE | P | β^S^ |  | β**^A^** | SE | P | β^S^ |
| X (Hypertension) | a | 0.725 | 0.117 | <0.001 | 0.127 | c’ | 0.418 | 0.089 | <0.001 | 0.087 |
| M (Sleep disturbance) |  | - | - | - |  | b | 0.364 | 0.050 | <0.001 | 0.434 |
|  |  | R^2^= 0.002 | | | | | R^2^= 0.190 | | | |
|  |  | F(1, 19136)= 38.174, P<0.001 | | | | | F(2, 19135)= 2245.302, P<0.001 | | | |

β^A^= unstandardized beta

β^S^= standardized beta
